# Partial Facetectomy for Lumbar Foraminal Stenosis

**DOI:** 10.1155/2014/534658

**Published:** 2014-07-08

**Authors:** Kevin Kang, Juan Carlos Rodriguez-Olaverri, Frank Schwab, Jenifer Hashem, Afshin Razi, Jean Pierre Farcy

**Affiliations:** ^1^Maimonides Medical Center, Department of Orthopaedic Surgery, 927 49th Street, Brooklyn, NY 11219, USA; ^2^New York University Hospital for Joint Diseases, New York, NY 10016, USA

## Abstract

*Background*. Several different techniques exist to address the pain and disability caused by isolated nerve root impingement. Failure to adequately decompress the lumbar foramen may lead to failed back surgery syndrome. However, aggressive treatment often causes spinal instability or may require fusion for satisfactory results. We describe a novel technique for decompression of the lumbar nerve root and demonstrate its effectiveness in relief of radicular symptoms. *Methods*. Partial facetectomy was performed by removal of the medial portion of the superior facet in patients with lumbar foraminal stenosis. 47 patients underwent the procedure from 2001 to 2010. Those who demonstrated neurogenic claudication without spinal instability or central canal stenosis and failed conservative management were eligible for the procedure. Functional level was recorded for each patient. These patients were followed for an average of 3.9 years to evaluate outcomes. *Results*. 27 of 47 patients (57%) reported no back pain and no functional limitations. Eight of 47 patients (17%) reported moderate pain, but had no limitations. Six of 47 patients (13%) continued to experience degenerative symptoms. Five of 47 patients (11%) required additional surgery. *Conclusions*. Partial facetectomy is an effective means to decompress the lumbar nerve root foramen without causing spinal instability.

## 1. Introduction

Lateral spinal root stenosis is a common condition occurring in 8 to 11% of surgical cases of lumbar degenerative disease [1]. With lumbar spondylosis, hypertrophy of the superior facets, buckling of the ligamentum flavum, disc bulge, protrusion of the annulus fibrosus, and osteophyte formation can lead to impingement of the exiting nerve root [2, 3]. A decrease in intervertebral disk height can also cause narrowing of the neural foramen and is frequently the etiology of lumbar radicular symptoms.

Impingement has also been implicated as a cause of failed back surgery syndrome due to unrecognized or possibly inadequate treatment of foraminal stenosis [4]. Therefore, it is an important pathology to recognize both clinically and radiographically in the treatment of a patient with radicular pain.

The preferred surgical treatment to relieve the compression on the exiting nerve root has not been established. Several different techniques to “decompress” the foramen have been described including foraminotomy, facetectomy, partial pediculectomy, fusion, distraction instrumentation, and posterior lumbar interbody fusion. We report the use of partial facetectomy for lumbar foraminal stenosis.

Indications for this procedure include patients without instability or central canal stenosis. The use of this procedure is precluded in patients with associated central or lateral stenosis. It is used only in cases of isolated foraminal stenosis in patients with lumbar radiculopathy unresponsive to nonoperative treatment. Additionally, facet joints that have lateral translation indicating underlying subtle instability or facets that are horizontally oriented are most suitable for this technique. In these circumstances, this procedure can provide adequate decompression to address significant leg pain and paresthesias that have failed conservative management.

## 2. Surgical Technique

**Figure 1:**
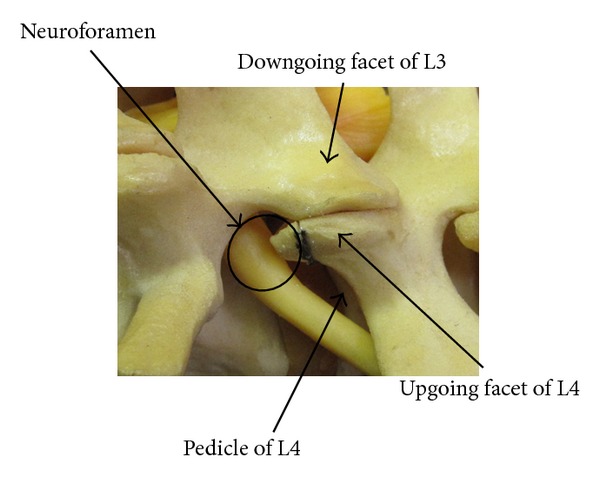
Sawbones representation of lumbar spinal anatomy. The borders of the foraminal space can be identified.

**Figure 2:**
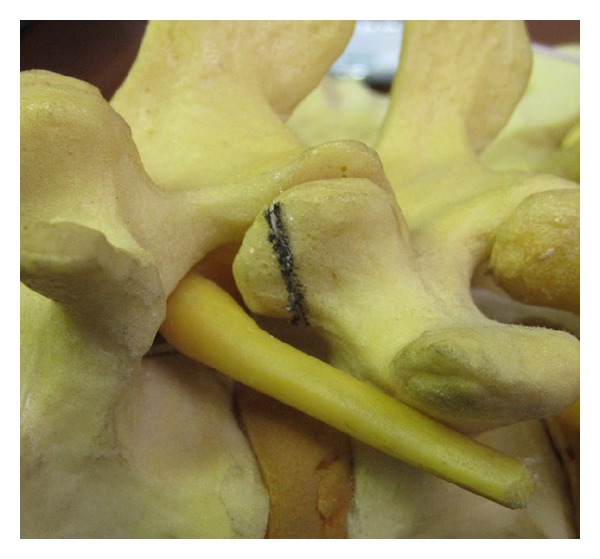
Planned partial resection of the upgoing facet.

**Figure 3:**
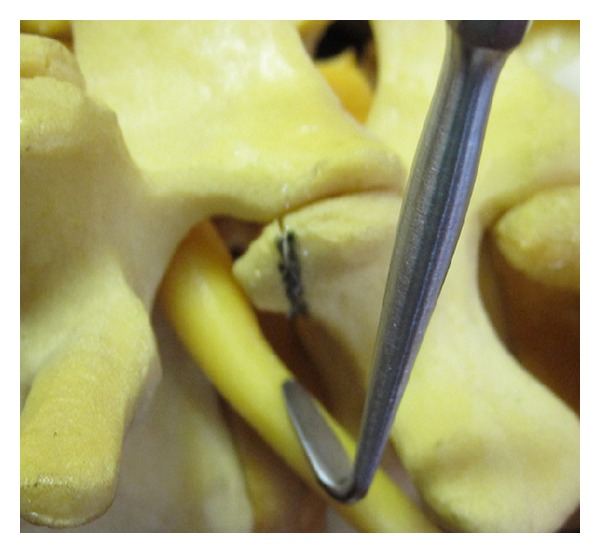
Dental instrument inserted to palpate the pedicle before osteotomy.

**Figure 4:**
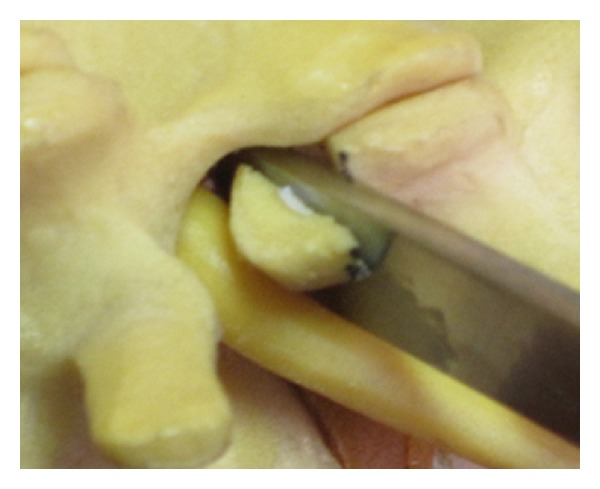
Osteotome performing the lumbar partial facetectomy.

**Figure 5:**
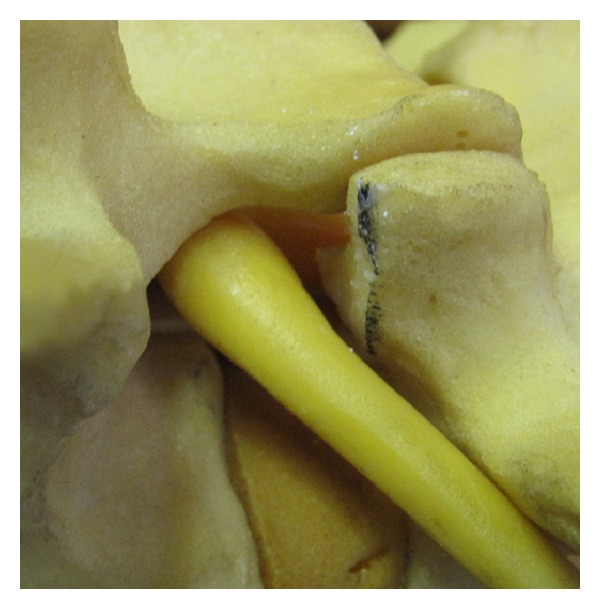
Neuroforamen after resection of bone.

**Figure 6:**
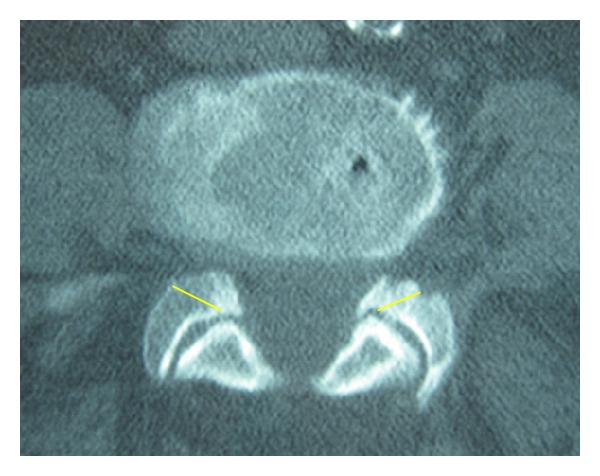
Axial CT scan image demonstrating foraminal stenosis and the planned bone resection.

**Figure 7:**
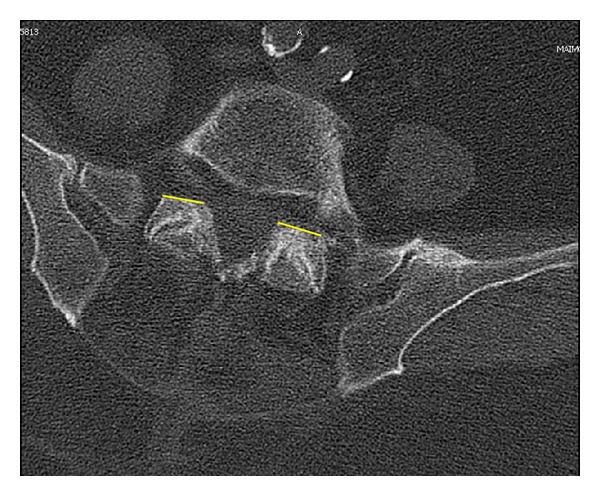
Postoperative axial CT scan image at the level of the facet joint demonstrating partial removal of the upgoing facets.

The foraminal space is delimited by the vertebral body, articular facets, and ligamentum flavum (Figure 1). The paramedian (Wiltse) approach is most suitable for both unilateral and bilateral stenosis. In the case of bilateral disease, two paramedian incisions are made. Using this approach allows the facet joints to be viewed at a 45-degree angle which facilitates this technique. The Wiltse approach requires the surgeon to dissect between muscle bellies in order to view the facet joints and have direct access to the joint line. Care must be taken to cause minimal damage to the muscle layer. The capsule is then incised over the superior part of the upgoing (superior) facet. The pedicle is palpated with a dental instrument to recognize and define the limit of the facet resection without pedicle injury (Figures 2 and 3). A stiletto osteotome is used to osteotomize the superior part of the facet (Figures 4 and 5). It is important that the stiletto slightly inclines medially so that the resection is limited to the joint capsule and the nerve root is protected. When the bone is cut, a curved curette can be inserted to pull and remove the bone fragments. Hemostasis can be achieved with bipolar cautery. Next, a ball pointed instrument can be used to palpate the foramen to assess for remaining causes of stenosis. Enlargement of the foramen can be accomplished employing a Kerrison rongeur to cut the protruding edges of the inferior facet and remove the attached redundant ligamentum flavum. Pre- and postoperative CT scan images can be seen in Figures 6 and 7.

## 3. Materials and Methods

47 patients who underwent partial facetectomy of the lumbar spine for foraminal stenosis between 2001 and 2010 were retrospectively reviewed. Prior to surgery, all patients were clinically evaluated and those with radicular symptoms were identified. Radiculopathy was defined as pain following a nerve root distribution which was exacerbated by standing and walking (neurogenic claudication).

Patients reported limitation of activity and inability to participate in recreational sports. 28 of 47 patients were unable to work and were on disability for anywhere between 2 and 28 months. On physical exam, they exhibited muscle weakness without atrophy as well as decreased reflexes. Electromyelogram (EMG) was also obtained prior to surgery to confirm signs of radiculopathy.

Conservative measures and noninvasive rehabilitation exercises were used in all patients for an average of 12 weeks prior to considering surgery. The level of stenosis was identified as L4-L5 in a majority of cases, with L3-L4 in 3 cases and L5-S1 in 2 cases.

Exclusion criteria for this study consisted of previous surgical intervention, instability of the lumbar spine documented on flexion/extension films, and central canal stenosis.

## 4. Results

The cohort consisted of 32 women and 15 men with an average follow-up of 3.9 years (range 2.5 to 12 years). The average age at the time of surgery was 59 (range 47 to 79). Follow-up information was obtained in 46 of 47 patients; one patient did not return for further evaluation after surgery. 27 patients reported no back pain and resumed their normal level of activity including recreational sports and full time work. Eight patients had occasional moderate back pain; however, their pain did not necessitate the use of analgesics and they were also able to return to work full time. Six patients experienced further degeneration after an average of 5.6 years, but did not seek further treatment. Five patients underwent a second surgery for additional decompression and fusion.

## 5. Discussion

Spinal stenosis is a broad term that encompasses all entities that decrease the space available for the spinal cord in the vertebral canal. According to Postacchini [5], three forms of degenerative lumbar spinal stenosis can be distinguished. Central stenosis refers to stenosis of the central part of the canal that often also involves the lateral corners. However, lateral stenosis involves the course of the nerve root from the thecal sac to the entrance of the intervertebral foramen. Foraminal stenosis is the final category and is caused by narrowing of the neuroforamen. Options for treatment and surgical intervention depend on the location of pathology and must be considered for satisfactory outcome.

Jenis and An [1] explained the pathoanatomy of foraminal stenosis. Lumbar spondylosis leads to the loss of intervertebral disk height and foraminal stenosis through the anterior and superior migration of the superior facet. The anteroposterior dimension of the neuroforamen decreases as the intervertebral disk height decreases. In addition, hypertrophy of the ligamentum flavum and osteophyte formation exacerbates the compression. The craniocaudal dimension can become compromised by vertebral endplate osteophytes, bulging annulus fibrosus, or a herniated disk. The combination of the above mentioned degenerative changes causes circumferential narrowing of the space available for the exiting nerve root which potentially leads to back pain and radicular symptoms. We have demonstrated promising results by employing partial facetectomy to address the foraminal stenosis.

An advantage of our described technique is that it preserves the stability of the spine. Much of the stability in the lumbar spine is provided by the anterior annulus fibrosus as well as the anterior longitudinal ligament. Haher et al. have shown that these structures are a key to maintaining rigidity, especially in extension [6]. However, the facet joints seem to be critical in maintaining rotational stability. In contrast to complete facetectomy, when medial partial facetectomy is carried out, segment stability is not compromised [7]. As long as preexisting instability is ruled out, partial facetectomy can be safely carried out without the need for fusion or instrumentation.

Fusion is usually indicated for preoperative instability or deformity, instability caused intraoperatively, or associated low back pain caused by degenerative disk disease. Fusion can be achieved through several different techniques including interbody fusion, transpedicular instrumented posterolateral fusion, and posterolateral fusion in situ. Instrumentation can be used to apply a distracting force to increase the size of the neuroforamen. However, care must be taken to prevent alteration of natural lumbar lordosis.

Related techniques have been described in the literature with good results. Ahn et al. [8] reported a posterolateral percutaneous endoscopic technique where an endoscope was inserted into the neuroforamen and a bone reamer was used to undercut the overhanging superior facet. A laser was also used to remove remnant ligamentous material. However, there are potential risks associated with this technique. First, a laser is unlikely to remove all bone fragments, leading to an incomplete decompression. Also, the laser may destroy a part of the annulus fibrosus leaving an open portal where intervertebral disk material can be extruded. Lastly, the heat generated by the laser can be the cause of iatrogenic nerve injury. Another technique has been described by Hejazi et al. [9]. They report a combined transarticular lateral and medial approach using microscopic assistance for partial facetectomy. However, our description shows that a similar decompression can be achieved using a traditional approach and instrumentation.

Although they report a different technique, Tender et al. [10] share a similar goal of providing adequate foraminal decompression without destabilizing the spine. In their approach, unilateral resection of the pars interarticularis is performed. Their biomechanical study [11] showed no significant difference in rotational stability after unilateral pars resection. However, their study was limited in that the possibility of contralateral fractures existed and they only evaluated spinal stability in the acute setting. With greater stress on the contralateral pars [12], fracture may potentially lead to instability, spondylolisthesis, and other degenerative changes.

Partial facetectomy is a safe and effective surgical technique for lumbar foraminal stenosis employing the standard approach and without causing secondary spinal instability. Long term follow-up shows sustained clinical improvement of symptoms.

## References

[1] Jenis LG, An HS (2000). Lumbar foraminal stenosis. *Spine*.

[2] Hasegawa T, An HS, Haughton VM, Nowicki BH (1995). Lumbar foraminal stenosis: critical heights of the intervertebral discs and foramina. A cryomicrotome study in cadavera. *Journal of Bone and Joint Surgery A*.

[3] Shenouda EF, Gill SS (2002). Laminal fenestration for the treatment of lumbar nerve root foraminal stenosis. *British Journal of Neurosurgery*.

[4] Burton R, Kirkaldy-Willis W, Yong-Hing K (1981). Causes of failure of surgery on the lumbar spine. *Clinical Orthopaedics and Related Research*.

[5] Postacchini F (1999). Surgical management of lumbar spinal stenosis. *Spine*.

[6] Haher TR, O'Brien M, Dryer JW (1994). The role of the lumbar facet joints in spinal stability: identification of alternative paths of loading. *Spine*.

[7] Abumi K, Panjabi MM, Kramer KM, Duranceau J, Oxland T, Crisco JJ (1990). Biomechanical evaluation of lumbar spinal stability after graded facetectomies. *Spine*.

[8] Ahn Y, Lee SH, Park WM (2003). Posterolateral percutaneous endoscopic lumbar foraminotomy for L5-S1 foraminal or lateral exit zone stenosis. Technical note. *Journal of Neurosurgery*.

[9] Hejazi N, Witzmann A, Hergan K, Hassler W (2002). Combined transarticular lateral and medial approach with partial facetectomy for lumbar foraminal stenosis: technical note. *Journal of Neurosurgery*.

[10] Tender GC, Baratta RV, Voorhies RM (2005). Unilateral removal of pars interarticularis. *Journal of Neurosurgery: Spine*.

[11] Tender GC, Kutz S, Baratta R, Voorhies RM (2005). Unilateral progressive alterations in the lumbar spine: a biomechanical study. *Journal of Neurosurgery, Spine*.

[12] Wang JP, Zhong ZC, Cheng CK (2006). Finite element analysis of the spondylolysis in lumbar spine. *Bio-Medical Materials and Engineering*.

